# Editorial: Recent Advances in Voltage-Gated Sodium Channels, their Pharmacology and Related Diseases

**DOI:** 10.3389/fphar.2016.00020

**Published:** 2016-02-08

**Authors:** Mohamed Chahine, Jean-François Desaphy

**Affiliations:** ^1^Centre de Recherche de l'Institut Universitaire en Santé Mentale de QuébecQuébec City, QC, Canada; ^2^Department of Medicine, Laval UniversityQuébec City, QC, Canada; ^3^Section of Pharmacology, Department of Biomedical Sciences and Human Oncology, University of Bari Aldo MoroBari, Italy

**Keywords:** voltage-gated Na channels, patch-clamp techniques, channelopathies, cancer, local anesthetics, pain, auxiliary subunits, post-translational modifications

After the success of the first Research Topic on voltage-gated sodium channels (Desaphy and Chahine), we were encouraged to re-open the topic for submission. In this second issue, we publish new insights in VGSC gating mechanisms, the role of β1-subunit, the involvement of VGSC in pain, encephalopathy, and cancer, and the molecular mechanism of VGSC drugs.

In humans, nine genes encode VGSC α-subunits that are responsible for ion permeation and voltage-dependent gating (Chahine et al., 2008; Savio-Galimberti et al.). The α-subunits are composed of four homologous domains (DI–DIV), each with six α-helical transmembrane segments (S1–S6). Residues of the S6 segments are thought to line the internal pore vestibule and contribute to the binding site for local anesthetics (LA) and antiarrhythmic drugs. Although the concept of state-dependent drug binding is well accepted, the underlying molecular mechanism is not well understood. The prevailing view is that conformational changes in the binding site associated with the voltage-dependent activation and inactivation of channels enhance drug binding and stabilize channels in non-conducting states. To assess the aqueous accessibility of DIVS6, O'Leary and Chahine introduced cysteine residues in the cardiac Nav1.5 channel and examined their sensitivity to MTSET, a thiol-specific reagent (O'Leary and Chahine). The MTSET inhibition of these cysteine mutants was well-correlated with the steady-state availability of the MTSET-modified channels, suggesting a link between fast inactivation and MTSET inhibition. MTSET modification of I1770C mutant disrupted fast inactivation, in agreement with the suggested contribution of the intracellular end of DIVS6 to the inactivation gate binding site. These data indicate that the docking of the inactivation gate induces a localized conformational change that regulates the aqueous accessibility of residues situated near the C-terminus of DIVS6.

Four accessory β-subunits (β_1_–β_4_) can complex with the α-subunit (Brackenbury and Isom; Chahine and O'Leary). The β-subunits have a single membrane-spanning α-helix with a large extracellular N-terminal domain incorporating an immunoglobulin-like fold resembling cell adhesion molecules. Thus it was proposed that, besides the modulation of α-subunits, β-subunits may participate in cell–cell and cell–matrix adhesion. Baroni and Moran reviewed the neuronal and cardiac channelopathies caused by β1-subunit mutations, which the high interindividual variability of symptoms and the underlying molecular mechanisms are not yet fully understood (Baroni and Moran).

The VGSC α-subunits and partner proteins are also modulated by post-translational modifications including phosphorylation, glycosylation, ubiquitination, and methylglyoxal-mediated glycation (Laedermann et al.). These mechanisms and their role in inherited and acquired pain syndromes are discussed by Laedermann and collaborators, which may open new avenues in the development of analgesics.

Inherited pain syndromes associated with Nav1.7, Nav1.8, and Nav1.9 mutations, are only a few of the many human sodium channelopathies. Mutations of Nav1.4 cause skeletal muscle disorders; Nav1.5 is responsible for genetic heart diseases; and mutations in Nav1.1 and Nav1.2 are responsible for a spectrum of epileptic syndromes. In their review, Wagnon and Meisler describe how the recent use of exome sequencing in patients with early-onset epileptic encephalopathy have permitted the identification of de novo mutations of Nav1.6 causing this non-familial disorder (Wagnon and Meisler). The first functional studies of Nav1.6 mutants suggest either a gain or a loss of channel function, so further studies are needed to complete our understanding of the pathological mechanisms and to identify the best treatment.

Besides their canonical role in excitable cells, the expression of VGSC in non-excitable tissues calls our attention to other possible function. The last two decades have shown an increasing number of studies reporting the abnormal expression of VGSC in cancer cells. Roger and collaborators summarize these studies, highlighting the possible critical role of VGSC in promoting cell migration and invasiveness (Roger et al.). VGSC may thus appear as promising druggable targets in cancer. Martin and collaborators provide us with a systematic review of studies testing the effects of sodium channel blockers in breast, colorectal, and prostate cancer (Martin et al.). Although preclinical studies suggest some benefits of these drugs in inhibiting cancer progression, there is still little information regarding their therapeutic value in humans. The authors claimed the need for a standardization of future studies and outcome measures to address this important issue.

The available VGSC blockers are used as anticonvulsants, antiarrhythmics, analgesics, neuroprotectants, and antimyotonics (Camerino et al., 2008). Most of these drugs show overlapping molecular mechanisms and little selectivity among VGSC isoforms. Their therapeutic index is mainly determined by their preferential binding to open/inactivated channels, allowing a selective action in pathological overexcited tissues (Fozzard et al.). Several drugs used for other indications are also able to potently inhibit VGSC. Lazar and collaborators investigated the different pH sensitivity of many of these VGSC inhibitors, refining our current conception of the role of alkalization in drug potency (Lazar et al.). In agreement with a previous study (Morris et al.), they propose a major role of ligand-membrane interactions in determining potency and selectivity of sodium channel blockers.

If, on the one hand, the “promiscuous” inhibition of VGSC by drugs known to primarily target other proteins may question their safe use, on the other, it was proposed that such activity may contribute to the therapeutic efficacy of some drugs. For instance, many analgesics inhibit VGSC at clinical concentrations, including tricyclic antidepressants and some opioids. In this topic, Carbonara and collaborators report the effects of new sumatriptan bioisosteres on neuronal Nav1.7 channels, a main contributor to pain transmission (Carbonara et al.). They identified new VGSC blockers with mixed serotoninergic 5HT_1D_ receptor agonism, which could represent interesting lead compounds for the synthesis of new anti-hyperalgesic drugs.

Altogether, these publications demonstrate that VGSC are still surfing on the crest of a wave with many current studies dealing with their intimate molecular gating mechanisms, their modulation by cell signaling processes, the genotype/phenotype relationship in sodium channelopathies, the exploration of novel function in non-excitable cells, and the search for new drugs. We are confident that new important VGSC findings will be published in a next future. Will this prompt us to re-open the topic again?

## Author contributions

All authors listed, have made substantial, direct, and intellectual contribution to the work, and approved it for publication.

### Conflict of interest statement

The authors declare that the research was conducted in the absence of any commercial or financial relationships that could be construed as a potential conflict of interest.
